# Is Alzheimer’s Also a Stem Cell Disease? – The Zebrafish Perspective

**DOI:** 10.3389/fcell.2018.00159

**Published:** 2018-11-23

**Authors:** Caghan Kizil, Prabesh Bhattarai

**Affiliations:** ^1^German Center for Neurodegenerative Diseases (DZNE) Dresden, Helmholtz Association, Dresden, Germany; ^2^Center for Regenerative Therapies Dresden, Cluster of Excellence, Technische Universität Dresden, Dresden, Germany

**Keywords:** zebrafish, Alzheimer’s disease, neural stem/progenitor cells, regeneration, neurogenesis

## Abstract

Alzheimer’s disease (AD) is the most common neurodegenerative disease and is the leading form of dementia. AD entails chronic inflammation, impaired synaptic integrity and reduced neurogenesis. The clinical and molecular onsets of the disease do not temporally overlap and the initiation phase of the cellular changes might start with a complex causativeness between chronic inflammation, reduced neural stem cell plasticity and neurogenesis. Although the immune and neuronal aspects in AD are well studied, the neural stem cell-related features are far less investigated. An intriguing question is, therefore, whether a stem cell can ever be made proliferative and neurogenic during the prevalent AD in the brain. Recent findings affirm this hypothesis and thus a plausible way to circumvent the AD phenotypes could be to mobilize the endogenous stem cells by enhancing their proliferative and neurogenic capacity as well as to provide the newborn neurons the potential to survive and integrate into the existing circuitry. To address these questions, zebrafish offers unprecedented information and tools, which can be effectively translated into mammalian experimental systems.

## The Re-Rise of Stem Cell Aspect for Neurodegenerative Diseases

Stem cells are the main reservoir for production of new cells. Understanding the basic biology of how stem cells are specified, maintained and regulated has been an exciting focus of research for many decades. Yet, there are still missing pieces especially on how stem cells could be utilized for neurodegenerative diseases (Jebelli et al., 2015; Tincer et al., 2016; Wyss-Coray, 2016). Stem cells offer great promises for medicine, as they can be the golden way to a “regenerative therapy” (Doetsch and Scharff, 2001; Lopez-Toledano and Shelanski, 2004; Rodriguez and Verkhratsky, 2011; Kizil et al., 2012b; Gage and Temple, 2013; Hong et al., 2014; Lilja et al., 2015). By using the endogenous stem cells, tissue loss could be reverted or the integrity of the existing tissues could be enhanced. Such outcomes would have ramifications in several human diseases but possibly among the most interesting is neurodegeneration (Tincer et al., 2016; Wyss-Coray, 2016). Indeed, since the generic term of “neurodegeneration” denotes the state of losing cells of the nervous system and in particular the neurons, transplantation of stem cells into the brain to get more neurons produced from these stem cells were one of the first treatment options (Dantuma et al., 2010; Mu and Gage, 2011; Rodriguez and Verkhratsky, 2011; van Tijn et al., 2011; Ager et al., 2015; Lee et al., 2015; Tincer et al., 2016; Wyss-Coray, 2016; Espuny-Camacho et al., 2017), for instance, injection of fetal NSCs for treating Parkinsonism (Baetge, 1993; Dunnett et al., 1997; Svendsen et al., 1997; Brundin and Bjorklund, 1998; Studer et al., 1998). These efforts could not gain spotlight as the transplanted stem cells or progenitors could not survive or could not form the desired cell types. For a couple of decades, the main focus in neurodegenerative diseases has been to prevent the neuronal death and synaptic failure (Carter and Lippa, 2001; Selkoe, 2003; Iqbal et al., 2016; Wyss-Coray, 2016). In Alzheimer’s disease (AD) – where the main culprit of the pathology is accumulation of Amyloid plaques and neurofibrillary tangles that lead to the loss of mostly cholinergic innervations in the brain – preventing the loss of synaptic degeneration and reduction in the neurotransmitter acetylcholine was prioritized as a therapy option (Fischer et al., 1987; Tuszynski et al., 1990; Nagele et al., 2002; Park et al., 2012; Gu et al., 2015). Several current drugs on the market for AD are blockers of the enzyme choline acetyltransferase, which degrades the cholinergic neurotransmitters in the brain. These drugs also failed to cure the disease despite causing meager slowdown in the cognitive decline in Alzheimer’s patients (Schneider et al., 2014). Similarly, physically destroying the plaques causes a cognitive advantage while does not fully restore the disease-associated symptoms (Takeda and Morishita, 2015). All these hypotheses and failures tell us a lesson: Alzheimer’s is not only a neuronal disease but also a complex mixture of malfunctioning in various cell types. An array of different cell types was implicated in the onset and progression of AD (De Strooper and Karran, 2016). These include changes in immune components (Amor et al., 2010; Heneka et al., 2015; Heppner et al., 2015), neurovascular niche (Kirkitadze et al., 2002; De Strooper and Karran, 2016), NSCs (Tong et al., 2015; Tincer et al., 2016), astrocytes (Attems and Jellinger, 2014; Lian and Zheng, 2016), and oligodendrocytes (Bartzokis, 2011; Ettle et al., 2016), suggesting a multifactorial influence on the initiation of AD. It can even be hypothesized that the loss of neurons – which is relatively a late symptom of the disease – might be the consequence of the yet-elusive real cause. When we generate a temporal onset of various symptoms of AD – mostly in animal models – we see that the first changes in the brain are the deterioration of the immune system balance, gliotic response from astrocytes and reduction in neural stem cell proliferation (Aguzzi and Haass, 2003; Selkoe, 2003; Blennow et al., 2006; Harman, 2006; Chai, 2007; Arendt, 2009; Hardy, 2009; Huang and Mucke, 2012; De Strooper and Karran, 2016; Dzamba et al., 2016; Tincer et al., 2016). As Amyloid deposition and neurofibrillary tangles occur, an inflammatory reaction manifests and becomes chronic in time. Concomitant to this reaction, NSCs also reduce their proliferation rate and produce less neurons long before the myelin breakdown, synaptic degeneration and neuronal cell death manifest (Demars et al., 2010; Tincer et al., 2016). Therefore, it is a plausible hypothesis to think that the inflammatory environment is negatively affecting the brain homeostasis in Alzheimer’s conditions not only by eliciting a chronic inflammation that is detrimental for synapses on its own but also by reducing the capacity of the brain to produce more neurons – an ability that could have been utilized to replace the lost neurons. These questions seem to have opened a wide research realm focusing on the role of immune system in AD (Akiyama et al., 2000; Heneka et al., 2005, 2015; Wyss-Coray, 2006; Amor et al., 2010; Aguzzi et al., 2013; Heppner et al., 2015; Kizil et al., 2015). Many reports documenting the effects of inflammation on AD pathology and the role of immune cells on the progression of the disease emerged. It is quite likely that coming years will bring important paradigm shifts in the relationship of immune system and the AD. However, a largely overlooked phenomenon in this context is the NSCs. Can NSCs and neurogenesis be the key to the cure for neurodegeneration? (Ziabreva et al., 2006; Waldau and Shetty, 2008; Taupin, 2009; Dantuma et al., 2010; Rodriguez and Verkhratsky, 2011; Tincer et al., 2016). This is where zebrafish could contribute to the answer of this provocative question.

## Zebrafish and the Hope for Stem Cell-Based Regenerative Therapies

No existing model for AD recapitulates the full spectrum of the disease, and existing mouse models are not exceptions (LaFerla and Green, 2012). These models can be considered at best the tools to study the early onset stages of Alzheimer’s (De Strooper and Karran, 2016). Although mouse models provided invaluable information on the pathology of AD, these mammalian models are not ideal to study “regeneration” as they do not have regenerative ability at first place (Goss, 1991). Zebrafish, an animal model that can regenerate its neurons offers unprecedented hope for restoring lost neurons in AD (Kizil et al., 2012b; Cosacak et al., 2015; Tincer et al., 2016; Kizil, 2018).

Mammals fail to regenerate amputated limbs, cardiac tissue, brain or spinal cord due to their restricted and limited regenerative potential (Tanaka and Ferretti, 2009; Poss, 2010). Current studies focus to improve methods or develop novel approach that can induce regenerative programs into the mammalian systems (Antos and Tanaka, 2010; Gemberling et al., 2013; Cosacak et al., 2015). One approach is to induce regeneration by activating endogenous regeneration programs. Zebrafish could serve as a model to understand those molecular cues as many “regeneration” programs were identified in zebrafish and they serve as interesting candidates toward this aim (Raya et al., 2003; Zupanc, 2008; Kizil et al., 2009, 2012a,b,c; Millimaki et al., 2010; Kyritsis et al., 2012; Diotel et al., 2013; Berberoglu et al., 2014; Cosacak et al., 2015; Alunni and Bally-Cuif, 2016; Bhattarai et al., 2016; Katz et al., 2016; Mokalled et al., 2016; Kizil, 2018; Than-Trong et al., 2018). Hence, the remarkable feature of regeneration in zebrafish deserves a closer attention for translational ramifications.

The exciting yet provocative argument of “zebrafish can teach us” could be challenged from another perspective: it could also be argued that the reduced capability of regeneration in rodents makes them better models as they are closer to the human situation. It is surely true that a model, which is as close as possible to human condition, would be ideal to work out reductionist aspects of a disease and indeed the mouse models offered invaluable knowledge on AD pathology. However, regenerating organisms endow a novel perspective of stem cell plasticity and regenerative ability that might be harnessed for therapeutic ramifications in humans but may not be investigated in mammalian systems. If nature has evolved a set of molecular programs that enable regenerative output of NSCs in AD conditions, zebrafish and other regenerating organisms but not mammals could teach us these programs. In the long run, those programs must be tested in mammals to investigate if they are evolutionarily conserved and whether they are sufficient to elicit a stem cell response similar to that of zebrafish. This could be the step where zebrafish could come in handy: identification of naturally occurring “candidate” programs that might underlie a regenerative touch to the old problem of AD. It is also necessary to mention that the nature of regenerative ability and why it is lost evolutionarily in mammals are still unknowns. Therefore, the applicability of the knowledge from zebrafish to humans needs further studies, which will shed more light onto the extent of parallelism between mammals and zebrafish in disease conditions.

## Neural Stem Cells and Neuronal Regeneration

Mammalian nervous system contains NSCs that give rise to newborn neurons during development as well as adulthood (Doetsch et al., 1999; Gage, 2000; Conti and Cattaneo, 2010; Gage and Temple, 2013). The ability of NSCs to form neurons however varies and is still controversial (Kronenberg et al., 2003; Galvan and Jin, 2007; Kempermann et al., 2008, 2018; Ernst et al., 2014; Magnusson et al., 2014; Urban and Guillemot, 2015; Magnusson and Frisen, 2016; Boldrini et al., 2018; Sorrells et al., 2018). During development, NSCs give rise to all neuronal subtypes (Gage, 2000; Temple, 2001; Doetsch, 2003; Kriegstein and Alvarez-Buylla, 2009; Hansen et al., 2010; Pacary et al., 2012; Urban and Guillemot, 2015). But, during the adulthood, the NSCs are restrictive and limited to fewer areas – the subventricular zone (SVZ) of the lateral ventricle and the dentate gyrus of the hippocampus (Doetsch and Scharff, 2001; Alvarez-Buylla et al., 2002; Spalding et al., 2013; Kempermann et al., 2018). Though constitutive neurogenesis occurs in these neurogenic regions, upon injury they fail to achieve neuronal repair due to lack of neurogenic inputs (Silver and Miller, 2004; Rolls et al., 2009; Costa et al., 2010). For instance, in case of mammalian traumatic injury model, there is absence of permissive environment for NSCs to react effectively.

Unlike mammals, zebrafish can successfully regenerate the injured part of its brain (Chapouton et al., 2007; Zupanc, 2008; Kroehne et al., 2011; Baumgart et al., 2012; Kishimoto et al., 2012; Kizil et al., 2012a,b,c; Kyritsis et al., 2012; Marz et al., 2012; Barbosa et al., 2015; Cosacak et al., 2015; Bhattarai et al., 2016; Kizil, 2018). This ability is possible because of the stem cell niches and the neurogenic regions that harbors proliferative neural progenitor cells (Adolf et al., 2006; Grandel et al., 2006; Chapouton et al., 2007; Kaslin et al., 2009). However, there is more to it. The regenerative ability after neuronal loss in zebrafish brain relies on the activation of specific molecular mechanisms that do not exist in normal homeostatic state or even during development of those structures (Zupanc, 2008; Kaslin et al., 2009; Fleisch et al., 2010; Kizil et al., 2012b; Cosacak et al., 2015; Alunni and Bally-Cuif, 2016; Kizil, 2018; Shimizu et al., 2018). There is still a long way to understand the complete picture that makes the zebrafish brain special, yet the path is quite promising. Can we understand in zebrafish how new neurons are made and can we harness this information for humans to effectively regenerate our brains when needed – for instance in AD?

## Addressing Stem Cell Potential in Alzheimer’s Disease Model in Adult Zebrafish Brain

**FIGURE 1:**
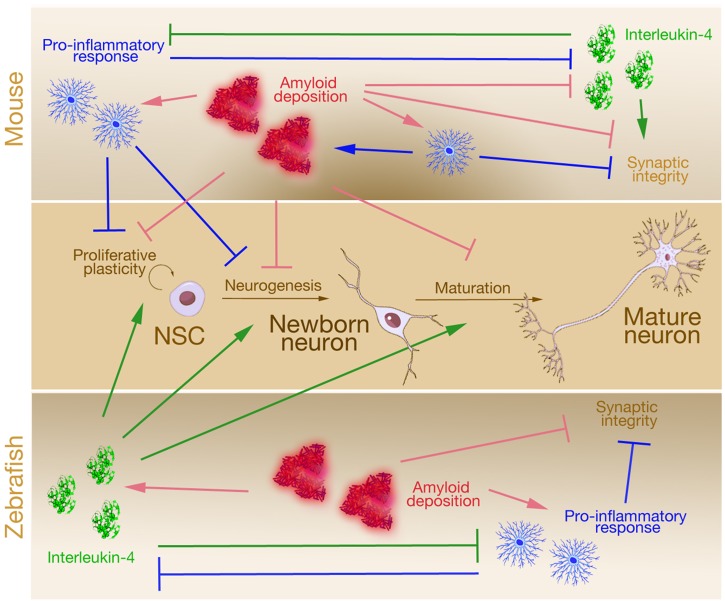
A simplified comparison of the effects of Alzheimer’s disease on neural stem cell plasticity in mouse and zebrafish. In mouse, Amyloid deposition initiates pro-inflammatory response that potentiates Amyloid toxicity that impairs neural stem cell proliferation, neurogenesis, neuronal maturation, and synaptic integrity. This chronic inflammation suppresses anti-inflammatory factor Interleukin-4, which is beneficial for neuronal survival and synaptic integrity. In zebrafish, although Amyloid deposition follows a toxicity cascade similar to that of the mouse (activation of pro-inflammatory response and hampered synaptic integrity), Amyloid also leads to induction of anti-inflammatory factor Interleukin-4, which enhances neural stem cell proliferation, neurogenesis, and neuronal maturation. The effects of Interleukin-4 counteracts synaptic degeneration and reduced neural stem cell plasticity.

One of the hallmarks of AD is accumulation of amyloid plaques that are made up of the short peptide Amyloid-beta42 (Aβ42) (Yang et al., 1995; Duff et al., 1996; Younkin, 1998). In mammals, plaques elicit chronic inflammation and together with the plaques lead to synaptic failure, reduced neural stem cell plasticity and neurogenesis (Figure 1). We recently developed a microinjection-based method to generate an Aβ42 model in adult zebrafish that displayed AD-like phenotypes (Bhattarai et al., 2016, 2017a,b). Aβ42 aggregation in adult zebrafish brain led to phenotypes reminiscent of human AD pathophysiology: neuronal death, inflammation, synaptic degeneration, memory and learning deficits. In addition, this model also induced regenerative response by activation of NSCs and subsequent neurogenesis to compensate the neuronal insult (Figure 1). Therefore, this Aβ42 toxicity model in adult zebrafish offers an opportunity to study the molecular mechanisms how NSCs can be activated to form neurons and induce regeneration in AD condition. Interestingly, this regenerative neurogenesis response upon Aβ42 in adult zebrafish brain was mediated by a crosstalk between the immune system and the NSCs via an unexpected mediator: Interleukin-4 (IL4), an anti-inflammatory cytokine (Figure 1). Although the role of IL4 in suppressing the inflammatory response and in turn relieving the suppressive effects of inflammation on the neural stem cell proliferation in mammalian Alzheimer’s models were known, the direct regulation of the inflammatory environment on NSCs – which are the only non-immune cell types that express the receptor for IL4 – was a novel finding. Even with known molecules, zebrafish could provide novel understanding and ideas on how crosstalk mechanisms between the neurodegenerative milieu and the NSCs in the adult zebrafish brain could induce regenerative response (Bhattarai et al., 2016; Kizil, 2018). These studies also proposed that neural stem cell activity might be key to a successful recovery from neurodegeneration.

## Can Alzheimer’s Be Treated With Increased Neurogenesis?

The role of neurogenesis in Alzheimer’s pathology and whether new neurons could really rescue the symptoms of Alzheimer’s is quite controversial and some researchers are skeptical toward this approach because the effects of Amyloid deposition on stem cell proliferation are beneficial or detrimental in a context dependent manner (Haughey et al., 2002; Lopez-Toledano and Shelanski, 2007; Diaz-Moreno et al., 2013; He et al., 2013; Lee et al., 2013; Bhattarai et al., 2016). Since neurogenesis cannot be equated with functional integration into the circuitry, the need to detect the effects of newborn neurons on circuit integrity has not been met sufficiently (Wen et al., 2004; Yamasaki et al., 2007; Blurton-Jones et al., 2009; Gomez-Nicola et al., 2014). However, when we scrutinize the course of manifestation of AD, we see that the NSCs are affected during the neurodegenerative conditions in all mammalian model systems tested: a progressive decline in neural stem cell pool during the course of neurodegeneration (Haughey et al., 2002; Ziabreva et al., 2006; Waldau and Shetty, 2008; Rodriguez and Verkhratsky, 2011; He et al., 2013; Martinez-Canabal, 2014; De Strooper and Karran, 2016; Dzamba et al., 2016; Tincer et al., 2016). But in case of Aβ42-mediated neurodegeneration in zebrafish, increased neuronal death was followed by increased proliferation of NSCs (Bhattarai et al., 2016, 2017a,b). Zebrafish brain reacted to neurodegeneration by utilizing neuro-inflammatory crosstalk to mediate the regenerative response. It indicates that the molecular mechanism regulating the regenerative response after amyloid-mediated neurodegeneration was pathology-induced plasticity response, and could be helpful to alleviate the symptoms of AD (Kizil, 2018). In fact, supporting evidence to this hypothesis came from comparative studies in a tissue mimetic 3D human NSCs plasticity assays and neuronal cultures as 3D systems are emerging as promising surrogates for human brain disease modeling (Justice et al., 2009; Haycock, 2011; Tang-Schomer et al., 2014; Zhang et al., 2014; Pasca et al., 2015; Ravi et al., 2015; Choi et al., 2016; Murphy et al., 2017; Papadimitriou et al., 2018). To test whether IL4 would act, similarly, in humans during AD – and therefore can be used as a regenerative paradigm, we developed an *in vitro* 3D culture system to grow mature human cortical neurons and networks from human NSCs (Papadimitriou et al., 2018). This system provides an in-vivo like environment including the essential components of the extracellular matrix, which are dynamically produced by the cultured cells and allows experimentation on a wide spectrum of human brain physiology: from neural stem cell plasticity to neuronal differentiation, from neuronal maturation to integration of neurons into existing networks. Adapting a glycosaminoglycan-based, cell-responsive hydrogel platform, we stimulated primary human neural stem cells (NSCs) from human cortex to form extensive neuronal networks *in vitro*. The 3D cultures exhibited neurotransmitter responsiveness, electrophysiological activity, tissue-specific extracellular matrix (ECM) deposition, and the expression of pro-neural genes and cortical neuronal markers that are undetectable in conventional 2D cultures. Importantly, those cultures formed from primary (human fetal) cortical cells, closely resemble the human physiology, which is critical for any disease modeling or therapeutic drug discovery efforts. The 3D cultures displayed a robust neural stem cell proliferation and neuronal differentiation, which is essential for a self-sustaining germinal niche of the human brain. After being formed *in situ*, our cultures express mature cortical neuronal markers showing a tissue-mimetic development (Papadimitriou et al., 2018). In this system, we modeled Amyloid toxicity as in adult zebrafish brain and found that the 3D culture system nicely recapitulated the major Alzheimer’s phenotypes such as the synaptic degeneration, loss of network connectivity, reduced neural stem cell proliferation and Tauopathies in a highly reproducible manner (Papadimitriou et al., 2018). Interestingly, treatment with IL4 under high Amyloid burden restored the neural stem cell proliferation, neurogenesis, network formation and functional integration of neurons into the existing circuitry, suggesting that increasing the neurogenesis in Alzheimer’s conditions could rescue the symptoms and might be a plausible way to cure this disease. In fact, a recent *in vivo* study found that increasing adult neurogenesis in Alzheimer’s model of mice increases the cognitive abilities and generated a healthier brain microenvironment in AD conditions (Choi et al., 2018), suggesting that the role of neurogenesis in conjunction with inflammation is a charming research realm in AD.

Notwithstanding with the ease of charting this interaction, realizing an immune-stem cell crosstalk in human brains that will lead to a real recuperation seems like a sci-fi novel. However, we know quite a bit on how inflammation is affecting the AD brain (Akiyama et al., 2000; Sastre et al., 2006; Amor et al., 2010; Glass et al., 2010; Aguzzi et al., 2013; Heneka et al., 2013, 2015; Heppner et al., 2015). Chronic phase of inflammation impinges on stem cell plasticity and synaptic integrity while resolution of inflammation provides a relief on the inflammatory burden and affected cell types may regain their potentials. An example of this regulation pertaining to our findings is the effects of Interleukin-4. After experimental models of inflammation, microglial dynamics were shown to be regulated by Interleukin-4 (e.g., pro-inflammatory cytokine release and the extent of initial inflammatory response) and this had an effect on neurogenesis dynamics and neuronal activity (e.g., long term potentiation in hippocampus, neural stem cell proliferation and neuroprotection) (Maher et al., 2005; Nolan et al., 2005; Lyons et al., 2007, 2009; Clarke et al., 2008; Nunan et al., 2014; Barrett et al., 2015). These “beneficial” effects of IL4 was considered to be because of its anti-inflammatory roles. However, in mouse brains, a direct interaction between anti-inflammatory factors and NSCs was not shown. In zebrafish and 3D cultures of human brains, on the other hand, IL4 seems to be directly affecting neural stem/progenitor cells by enhancing their neurogenic output (Bhattarai et al., 2016; Papadimitriou et al., 2018). This proposes an alternative approach to neuroinflammation research where we may need to decouple the microglial inflammation dynamics and direct interaction of immune factors with NSCs, which may be a collateral by-stander effect. In one hypothetical scenario, we may need to investigate which molecules partake in the direct crosstalk between immune system and NSCs in zebrafish and see whether those molecules are able to activate NSCs directly in mammals. Given that even though an immune-related factor would be available in AD brains, its effect is limited to those cells that can receive the signal. The by-stander effects of immune factors could be used to design a stage-specific modulation of NSCs in disease conditions. By a hypothetical scenario, we can appreciate why the immune-related signaling in neuronal compartment and in NSC niche can give us alternative treatment options in humans. For example, in a scenario, an immune factor could turn out to be beneficial for NSC plasticity in AD conditions, but this molecule would be an anti-inflammatory factor (e.g., IL4). Therefore, this factor would prevail only when there is a resolution of inflammation, which is not the case in AD. Therefore, the human NSCs would not be able to increase their proliferation simply due to the stage of the disease (they could otherwise do). Then, a drug can be designed to activate the immune-type signaling in NSCs regardless of the inflammation conditions and this can help elicit a neurogenic contribution from NSCs even if the inflammation is not resolved. When combined with strategies to increase the survival of newborn neurons, such a “nudge” on NSCs could contribute to the remedy of the disease, which could otherwise not happen naturally. Therefore, understanding the direct interaction of immune system with NSCs by using zebrafish and other appropriate models is important to establish deeper knowledge on the crosstalk between various cell types and NSCs. Additionally, activating the neural stem cell proliferation and neurogenesis in AD conditions must definitely be re-visited as an effective way of tackling this horrendous disease. The neural stem cell aspect of the AD could also provide us new ways for clinical therapies and may help to overcome the inefficient drug discovery efforts for Alzheimer’s so far.

## Limitations and Promises Ahead

Although zebrafish could be an excellent tool from which we could understand how NSCs could be utilized to revert the symptoms of AD, there are experimental and physiological limitations we have to consider (Table 1). Zebrafish is a vertebrate and has evolutionary similarities to humans; however, it is still different than the human brains in terms of complexity, molecular structure, and physiology. Given that even mouse models of Alzheimer’s cannot be perfect surrogates for human disease, it would be naive to assume that zebrafish brain would fully recapitulate the AD in human brains. This is an aspect where the disease models could be refined in fish and could be made more compatible with human situation. By doing so, zebrafish could also be in part used for early phase pre-clinical studies to test drug efficiency. Additionally, the neurodegenerative disease models should be diversified in zebrafish in order to match the versatility of disease causing proteins. A future perspective for AD modeling in zebrafish could be to generate transgenic animals that display a more chronic and steady accumulation of disease hallmarks that persist throughout the adult stages. Several examples of those efforts are emerging (Malaga-Trillo et al., 2011; Xi et al., 2011; Schmid and Haass, 2013; Cosacak et al., 2017; Lopez et al., 2017; Kizil, 2018). Additionally, using comparative mammalian assays such as organoids or 3D culture systems (Choi et al., 2014, 2016; Fatehullah et al., 2016; Mansour et al., 2018; Papadimitriou et al., 2018) could be a way to check the stringency of conclusion from zebrafish as to whether or not they would hold true in mammalian brains.

**Table 1:** Comparison of zebrafish and rodent models in Alzheimer’s disease research.

Zebrafish	Rodents
**Zebrafish advantegeous over rodents**	
Amyloid-mediated neuronal death	No neuronal death
Neuroregenerative capacity	No neuroregenerative capacity
Stem cell plasticity for neurogenesis	Stem cells reduce plasticity and neurogenesis
Cost efficient generation and maintenance	Expensive generation and maintenance
High number of animals testable	Limited number of animals testable
3R strategies developed	3R strategies to be developed
**Zebrafish and rodents equal**	
Synaptic degeneration	Synaptic degeneration
Cognitive decline with Amyloidosis	Cognitive decline with Amyloidosis
Genetic tools available	Genetic tools available
Does not reflect the entire biology of the human disease	Does not reflect the entire biology of the human disease
**Rodents advantegeous over zebrafish**	
Non-mammalian physiology	Mammalian physiology
Need for adaptation to preclinical studies	Suitable for preclinical studies
Limited number of models expressing disease-related proteins	Variety of models expressing disease-related proteins

Despite its disadvantages listed above, zebrafish holds up well with the handiness of the rodent models of AD in several aspects such as the diversity of genetic tools and the ability of modeling disease hallmarks such as synaptic degeneration and cognitive decline (Table 1). Nevertheless, neither mouse models nor zebrafish models can recapitulate the whole pathophysiological biology of AD as in human brains, which suggests that those models are useful insofar as their strengths in particular aspects were found. For instance, zebrafish is quite advantageous over rodents in many aspects (Table 1). These include (1) the pathological outcomes that resemble the human brain such as the ability of Amyloid depositions to lead to neuronal death, (2) regenerative ability owing to the capacity of NSCs to respond to tissue loss by enhanced plasticity and neuro-regenerative outcome, (3) cost of experimental studies, (4) number of animals that can be tested in a laboratory setting, and (5) the availability and possibility of 3R-friendly experimentation schemes.

## Outlook

Although zebrafish lags behind the mammalian models in certain aspects, it already outperforms in many (Table 1). For instance, stem cell biology in zebrafish offers unprecedented information on the molecular programs that enable stem cell-based regeneration. Many research reports contributed to understanding of how NSCs in adult zebrafish brain function, are affected by external cues and respond to loss of neurons through recruitment of diverse signaling pathways including Notch, Wnt, Fgf, Bmp, and chemokine signaling (Mueller et al., 2004; Adolf et al., 2006; Grandel et al., 2006; Chapouton et al., 2007, 2011; Pellegrini et al., 2007; Zupanc, 2008; Diotel et al., 2010, 2013; Kroehne et al., 2011; Rothenaigner et al., 2011; Baumgart et al., 2012; Coolen et al., 2012, 2013; Kishimoto et al., 2012; Kizil et al., 2012a,b,c; Kyritsis et al., 2012; Marz et al., 2012; Alunni et al., 2013; Salta et al., 2014; Barbosa et al., 2015; Rodriguez Viales et al., 2015; Than-Trong and Bally-Cuif, 2015; Alunni and Bally-Cuif, 2016; Bhattarai et al., 2016; Kizil, 2018; Shimizu et al., 2018; Than-Trong et al., 2018). This large repertoire of knowledge will be instrumental in comparing the neuro-regenerative aptitude of zebrafish NSCs to human conditions and would help to find out how a successful proliferation-neurogenesis cascade could be elicited in mammals. Especially in the AD condition, which is the focus of this Perspective Article, such information could be instrumental and be a game-changer by providing an alternative approach to the disease mechanism and to its treatment (Figure 1). The role of neurogenesis in the manifestation of AD and its cure is rising to the spotlight again and zebrafish could offer valuable information on how our NSCs could be made “regenerative” using endogenous molecular programs and possibly by tweaking the immune system. Together with chemical/genetic screens, gene targeting and advances in research methodology; zebrafish stands out as an influential model that could drive preclinical findings toward novel clinically relevant discoveries.

## Author Contributions

CK formulated the perspective. PB and CK wrote the manuscript.

## Conflict of Interest Statement

The authors declare that the research was conducted in the absence of any commercial or financial relationships that could be construed as a potential conflict of interest.
